# Cellular angiofibroma of the vulva: a poorly known entity, a case report and literature review

**DOI:** 10.1186/s12907-016-0030-z

**Published:** 2016-06-04

**Authors:** Mouna Khmou, Najat Lamalmi, Abderrahmane Malihy, Lamia Rouas, Zaitouna Alhamany

**Affiliations:** Department of Pathology, Children’s Hospital Faculty of Medicine and Pharmacy, Mohammed V University Ibn Sina University Hospital, Rabat, Morocco

**Keywords:** Cellular angiofibroma, Vulva, Mesenchymal tumors, Histopathology, Immunohistochemistry

## Abstract

**Background:**

Cellular angiofibroma represents a newly described, site specific tumor. Histologically, CAF is a benign mesenchymal neoplasm characterized by two principal components: bland spindle cells and prominent small to medium-sized vessels with mural hyalinization. The indolent nature of the lesion is underscored by the uniformity of its constituent stromal cells, and their lack of nuclear atypia. Characterization by immunohistochemistry is helpful distinguishing Cellular angiofibroma from other mesenchymal lesions.

**Case presentation:**

We report the case of a 37-year-old woman, presenting with a painless nodule involving the vulva. This lesion had gradually increased in size; a simple excision was performed, and follow up was unremarkable. Gross examination showed a well circumscribed, firm tumor measuring 3× 3 × 2,5 cm. Histologically, the tumor was composed of uniform, short spindle-shaped cells, proliferating in an edematous to fibrous stroma and numerous small to medium-sized thick-walled vessels. A panel of immunohistochemical stains was performed, and confirmed the diagnosis of Cellular angiofibroma.

**Conclusion:**

In this report we aim to describe the clinical, pathological and immunohistochemical features of this rare entity through a literature review, and to discuss other vulvar mesenchymal lesions.

## Background

Cellular angiofibroma (CAF) is a rare benign mesenchymal lesion with a predilection for the genitourinary region. First described in 1997 [1], CAF is characterized by a spindle cell component and abundant small- to medium-sized thick-walled vessels [2]. Cases in males have been previously named “angiomyofibroblastoma-like tumor”. Besides two small series, cellular angiofibroma has been described only in isolated case reports, we found only 68 patients with genital CAF (Table 1) [3, 4]. To date, this last condition still remains a poorly known lesion that needs further investigations to closely define its clinical and pathological features.

**Table 1:** Summary of the literature review of vulvar CAF reported

Authors	Year	Age	Localisation	Treatment	Follow-up
Nucci et al. [1]	1997	50	Vulva	Complete excision	NA
		46	Left labia majora	Complete excision	NR, 19 months
		39	Right labia	Complete excision	NR, 12 months
		49	Labia	Complete excision	NA
Colombat et al. [25]	2001	37	Left labia majora	Complete excision	NA
Lane et al. [10]	2001	77	Left labia	Complete excision	NR, 12 months
Curry et al. [18]	2001	37	Clitoral hood	NA	NR, 15 months
Dufau et al. [16]	2002	53	Labia majora	NA	NA
Dargent et al. [9]	2003	46	Right labial region		NR, 19 months
		49	Lateral part of the clitoris.		NR, 7 months
McCluggage et al. [22]	2002	49	Left labia majora	Complete excision	Reccurence 6 months later
Iwasa et al. [3]	2004	49	Labia majora	Complete excision	NA
		39	Vulva	NA	NA
		46	Labia majora	Complete excision	NR, 16 months
		50	Vulva	Complete excision	Lost
		42	Vulva	Complete excision	NR, 75 months
		42	Perineum	NA	NA
		75	Vulva	Complete excision	Died of breast cancer
		41	Vulva	Complete excision	NR 54 months
		68	Vulva	Complete excision	NR, 17 months
		59	Labia majora	Complete excision	NR, 41 month
		49	Vulva	NA	NA
		37	Hymen Local	Excision + positive margins	NR, 24 months
		38	Vagina	NA	NA
		46	Vulva	Complete excision	NR, 35 months
		47	Labium majus	Complete excision	NR, 44 months
		47	Vulva	NA	NA
		48	Labium majus	Complete excision	NR, 8 months
		24	Vagina	NA	NR, 6 months
		58	Vagina	Complete excision	NA
		50	Vulva	Complete excision	NR, 6 months
		58	Vulva	Complete excision	NR, 9 months
		50	Vulva	NA	NA
W G McCluggage et al. [21]	2004	20	Not specified	Complete excision	NR, 20 month,
		25	Posterior vaginal introitus	Complete excision	NR, 3 months
		65	Left labia minora	Complete excision	NR, 12 months
		41	Left labia majora	Complete excision	NR, 4 months
		59	Right side of vulva	Complete excision	NR, 18 months
		32	Right labia	Complete excision	NA
Micheletti et al. [8]	2005	51	vulva	Complete excision	NR, 4 months
Kerkuta et al. [7]	2005	31	small left labial	Complete excision	NR, 10 month
Chen et al. [11]	2010	58	Vulva	Complete excision	NR, 75 months
		52	Vulva Local	Complete excision	Dead of carcinoma
		34	Vulva	Complete excision	NA
		32	Vulva	Complete excision	NA
		25	Vulva	Complete excision	NR, 42 months
		43	Vulva	Complete excision	NR, 2 months
		59	Vulva	Complete excision	NR, 14 months
		46	Vulva	Complete excision	NR, 4 months
		71	Vulva	Complete excision	NA
		39	Vulva	Complete excision	NR, 7 months
		46	Vulva	Complete excision	NA
Flucke et al. [4]	2011	41	Perineal	Complete excision	NA
		39	Vaginal introitus	Excision + positive margins	NR, 75 months
		50	Vulva	Excision + positive margins	NR, 55 months
		51	Labium majus	Marginal excision	NR, 66 months
		44	Labium majus	Complete excision	NA
		50	Vulva	Excision + positive margins	NA
		48	Vulva	Complete excision	NA
		42	Vulva	Complete excision	NA
		63	Clitoris	Excision + positive margins	NR, 38 months
		27	Labium majus	Marginal excision	NA
		42	Vulva	Complete excision	NR, 30 month
		46	Labium majus	Marginal excision	NA
		55	Vulva	Complete excision	NR, 12 months
		57	Vulva	NA	NR, 6 months
		47	Vulva	Excision + positive margins	NA
		39	Vaginal fornix	Marginal excision	NA
Present case	2015	37	Left labia majora	Complete excision	NR, 20 month

*NR* No Recurrence

*NA* information not available

We report a case of cellular angiofibroma, for which the clinical diagnosis was Bartholin’s glandular cyst.

## Case presentation

**Fig. 1:**
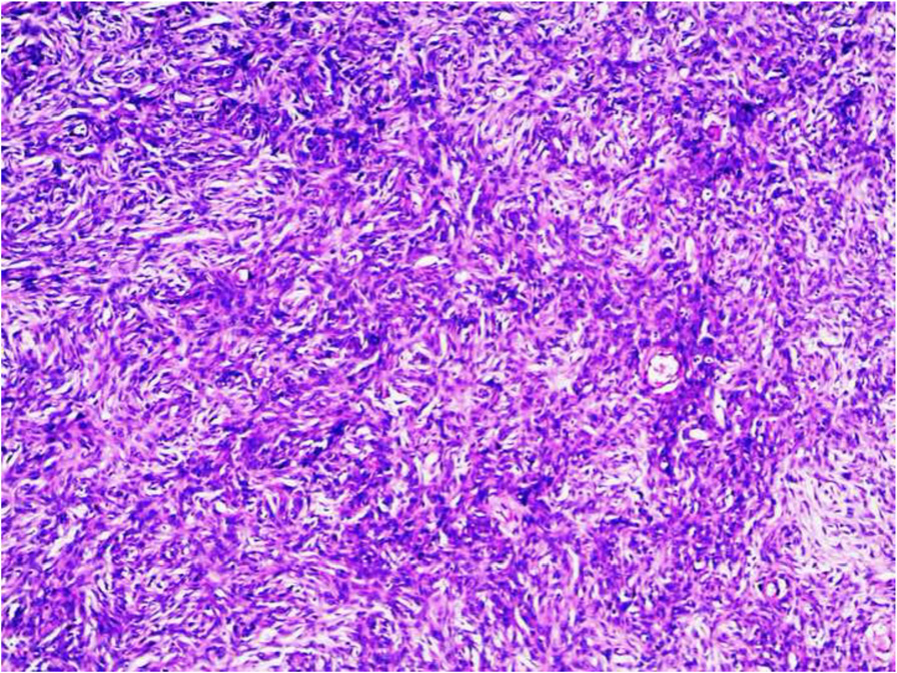
low-power view showing uniform, short spindle-shaped cells

**Fig. 2:**
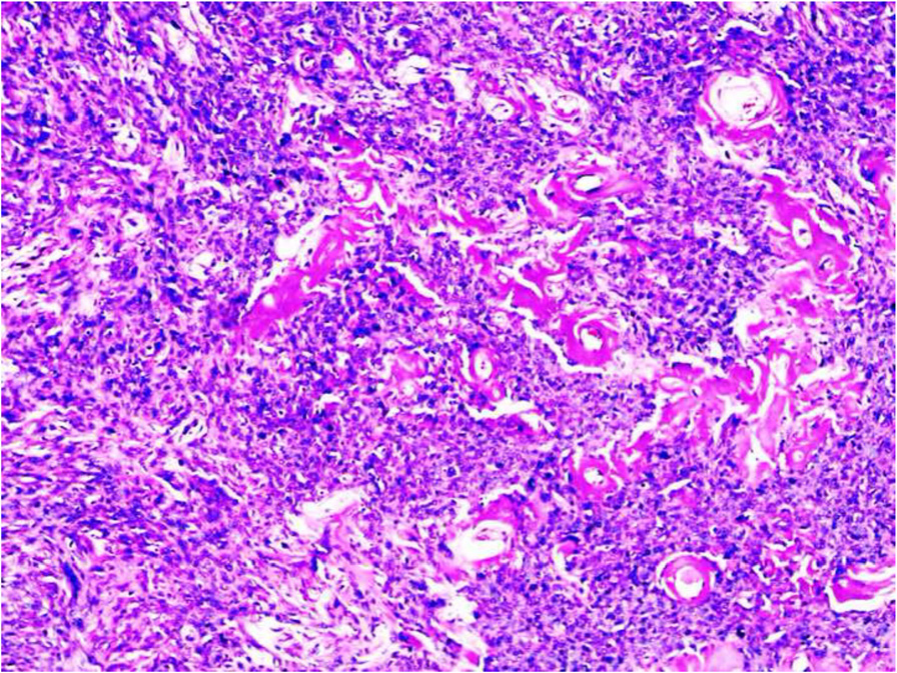
Numerous small to medium-sized with thick and hyalinized walls

**Fig. 3:**
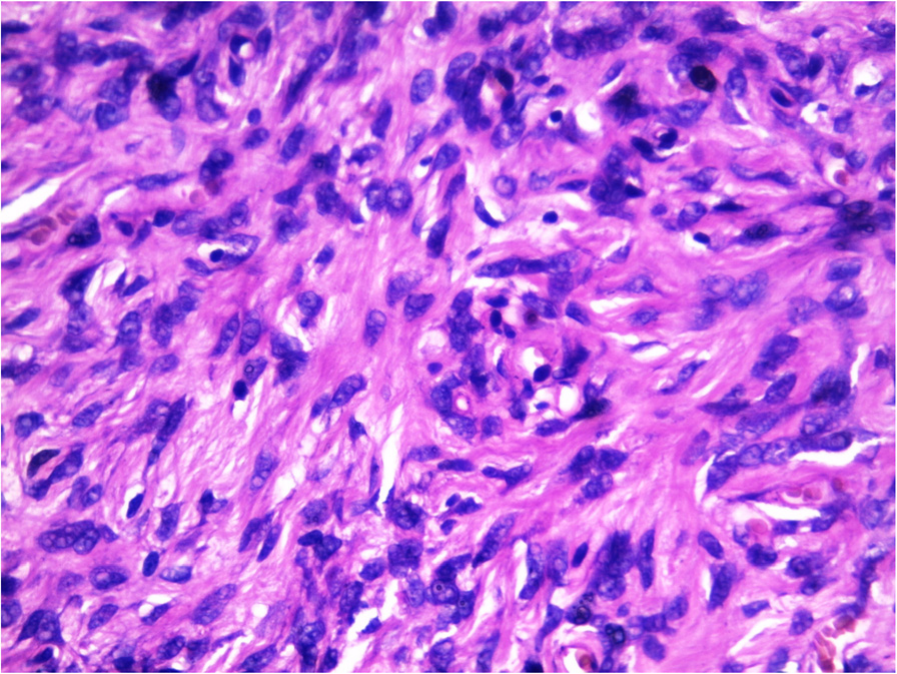
Bland spindle cells with uniform nuclei and pale indistinct cytoplasm

**Fig. 4:**
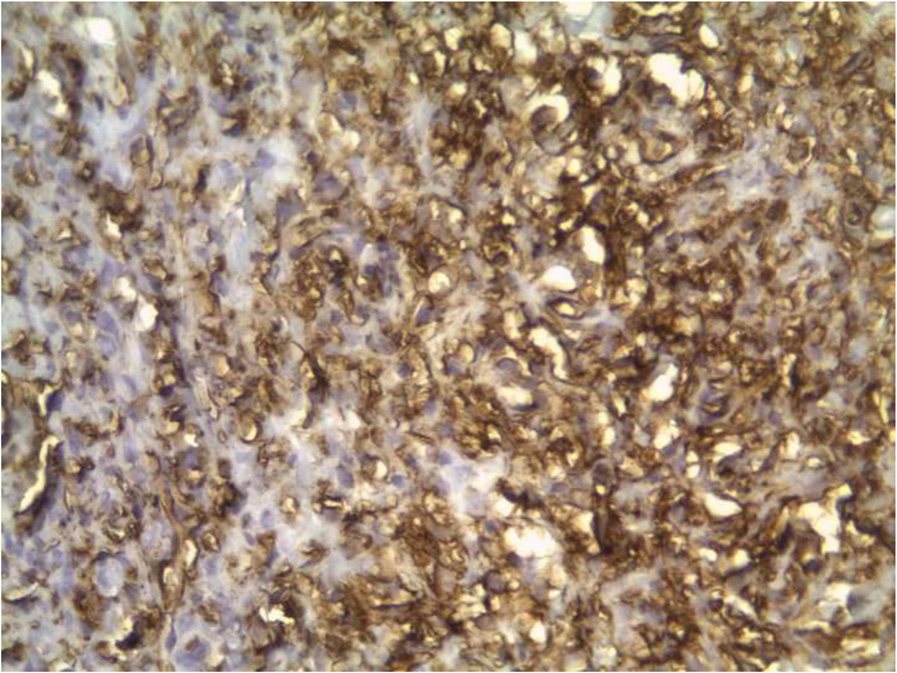
tumour cells exhibiting diffuse positivity with CD34

A healthy 37-year-old woman consulted for an asymptomatic vulvar nodule of 6 years duration. She was concerned because it had progressively enlarged over the last few months. There was no history of pain or bleeding. Local and colposcopic examinations revealed a 3,5 cm freely mobile non reducible nodule located in the left labia majora. Ultrasonography showed a superficial, well-demarcated, solid soft tissue tumor. A well circumscribed lesion measuring 3 cm in diameter was excised with a rim of normal tissue. Gross examination showed a well circumscribed, solid, whitish, glossy tumor measuring 3× 3 × 2,5 cm. Microscopically, the tumor was well circumscribed, surrounded by a fibrous pseudocapsule. On low-power examination, hypocellular and hypercellular areas, composed of uniform, short spindle-shaped cells, proliferating in an edematous to fibrous stroma (Fig. 1). Numerous small to medium-sized thick-walled vessels were also seen (Fig. 2). Mature adipocytes were noted in the periphery in small clusters. There was no necrosis and few or no mitotic figures (Fig. 3). Immunohistochemical staining was positive for vimentin, CD34 (Fig. 4), focally for actin, and negative for protein S-100, and desmin. These findings are consistent with the diagnosis of cellular angiofibroma. At 14 months postoperatively, the patient is doing well with no signs of recurrence.

## Discussion

Tumors primarily arising from the vulvo-vaginal area are relatively rare and they include soft tissue specific and non-specific tumors, as well as a spectrum of fibro-epithelial tumors [5, 6]. Cellular angiofibroma is an uncommon benign mesenchymal neoplasm, originally described in the genital region, and occurs equally in both genders [4]. A marked predilection for the vulva is observed [2], our review of the literature yielded 68 cases reported, involving the female genital tract (Table 1). Women are affected most often in the fifth decade, whereas males are mainly in the seventh decade [3]. Clinically, cellular angiofibroma is often mistaken for a Bartholin gland, labial, or submucosal cyst [7].

Etiopathologically, some authors suggested that these lesions are stem cell–derived, with a capacity for adipose and myofibroblastic differentiation in accordance with the influence of hormones, microenvironments, cytokines and growth factors [8].

Histologically, CAF is typically well circumscribed, composed of two principal components: bland spindle cells and prominent small to medium-sized vessels with mural hyalinization [3]. The spindle cells are arranged in short intersecting fascicles lying between short bundles of wispy collagen [9]. Hypocellular areas can be seen, often associated with stromal edema or hyalinization. Typically, significant pleomorphism and abnormal mitoses were absent [3]. The accompanying blood vessels tend to be thick-walled and even hyalinized [10]. Mature individual or small clusters of adipocytes can be present, most often located in the periphery of the lesion [2, 3]. Fletcher et al. recently have reported a study of 13 cases of cellular angiofibroma with atypia and sarcomatous transformation [11]. The sarcomatous component can show variable features (atypical lipomatous tumor, pleomorphic liposarcoma, and pleomorphic sarcoma). This phenomenon seems not to predispose to recurrence based on limited clinical follow-up available [2, 11].

Immunohistochemically, the tumor cells consistently are vimentin positive [9]. The expression of CD34 is seen in 60 % [3]. Characteristically, they do not express S-100 protein, actin, desmin, or EMA, although a discrete staining for the last three markers has been reported [3, 9]. Lastly, the tumor cells have been found to be estrogen (ER) and progesterone receptor (PR) positive. However, the significance of the positive estrogen and progesterone receptors in CAF is unknown [7]. In fact, a subset of mesenchymal cells of the distal female genital tract normally expresses estrogen and progesterone receptor and, the neoplastic cells arising from the vulva, may also show immunoreactivity for ER and/or PR [12]. Thus, ER or PR immunoreactivity cannot be used to distinguish CAF and its histological mimics [13]

No specific chromosomal abnormality is found in CAF, although cytogenetic analysis revealed, in a few reported cases, the loss of RB1 and FOXO1A1 genes due to the deletion of the 13q14 region [14]. This typical loss of genetic material is also shared by myofibroblastoma [15].

CAF, myofibroblastoma and angiomyofibroblastoma are usually considered as specific soft tissue tumors of the vulvo-vaginal area [16]. These tumors may show overlapping morphological, immunohistochemical and cytogenetic features, and thus differential diagnosis is mandatory [17, 15].

Clinically, the age of onset of CAF occurs approximately 10 years later in life than aggressive angiomyxoma, myofibroblastoma and angiomyofibroblastoma [18]. Histologically, aggressive angiomyxoma is poorly circumscribed, typically infiltrates adjacent soft tissue, and characterized by being composed of relatively uniform spindle cells, embedded in a myxoid matrix [10]. AMF is a benign tumor which belongs to the category of the “stromal tumors of the lower female genital tract”, together with cellular angiofibroma and myofibroblastoma [19]. It is characterized by the presence of multinucleate cells and epithelioid or plasmacytoid cells which tend to aggregate around blood vessels which are thin-walled [21]. However recent cytogenetic analyses have shown that only CAF and myofibroblastoma are genetically related lesions because angiomyofibroblastoma lacks 13q14 deletion [20].

Myofibroblastoma is composed of ovoid- to spindle- or stellate-shaped cells, arranged in a variety of architectural patterns and set in a finely collagenous stroma. Hyalinized blood vessels are a diagnostic clue helpful in distinguishing cellular angiofibroma from myofibroblastoma [15].

Based on morphological, immunohistochemical and cytogenetic analyses, it has been postulated that CAF and myofibroblastoma of the lower female genital tract are closely related lesions that form a continuous spectrum of a single entity with different morphologic presentations, likely arising from a common precursor mesenchymal cell [19].

Desmin seems to be a discriminating marker, as aggressive angiomyxoma, myofibroblastoma and angiomyofibroblastoma are positive for this antibody [3, 15].

Other neoplasms that are not specific to the vulva, such as solitary fibrous tumour, spindle cell lipoma, smooth muscle tumours, nerve sheath tumours, and perineurioma, also enter into the differential diagnosis [22].

Spindle cell lipoma is composed of brightly, eosinophilic ropy and refractile stromal collagen bands with fewer capillary-sized thin-walled vessels, compared with palely eosinophilic and wispy collagen fibers associated with numerous thick-walled vessels in CAF [3, 18]. Solitary fibrous tumor (SFT) can be differentiated by the presence of thin-walled branching vascular pattern that may be described as hemangiopericytoma-like vessels, and dense collagen bundles [12, 23]. SFT shows positivity for CD34, CD99, bcl-2, and ER and/or PR, and negativity for SMA and desmin [24].

Other mesenchymal lesions (schwannoma, perineurioma and leiomyoma) can be ruled out in accordance with the histology and immunohistochemistry [8].

CAF behaves in a benign fashion and local excision with clear margins is the treatment of choice. This lesion shows no tendency for metastasis based on the limited clinical follow-up available [2, 3, 7]. However, there is one case of recurrent CAF, reported by McCluggage et al., in which a 49-year-old woman had recurrent swelling develop at the site of the previous excision 6 months later [22]. Our patient is well without evidence of local recurrence 20 months after excision.

## Conclusions

CAF represents a rare distinct clinico-pathological condition, that pathologists should be aware of morphological variation (Atypia and Sarcomatous transformation) to prevent diagnostic errors and therefore an aggressive therapy. As far as we are aware, no case of metastatic CAF has been described.

## Abbreviations

AMF, Angiomyofibroblastoma; CAF, Cellular angiofibroma; EMA, Epithelial membrane antigen; ER, estrogen receptor; PR, progesterone receptor.
